# Interleukin-34 Restores Blood–Brain Barrier Integrity by Upregulating Tight Junction Proteins in Endothelial Cells

**DOI:** 10.1371/journal.pone.0115981

**Published:** 2014-12-23

**Authors:** Shijie Jin, Yoshifumi Sonobe, Jun Kawanokuchi, Hiroshi Horiuchi, Yi Cheng, Yue Wang, Tetsuya Mizuno, Hideyuki Takeuchi, Akio Suzumura

**Affiliations:** Department of Neuroimmunology, Research Institute of Environmental Medicine, Nagoya University, Furo-cho, Chikusa-ku, Nagoya, 464-8601, Japan; Kyushu University, Japan

## Abstract

Interleukin-34 (IL-34) is a newly discovered cytokine as an additional ligand for colony stimulating factor-1 receptor (CSF1R), and its functions are expected to overlap with colony stimulating factor-1/macrophage-colony stimulating factor. We have previously shown that the IL-34 is primarily produced by neurons in the central nervous system (CNS) and induces proliferation and neuroprotective properties of microglia which express CSF1R. However, the functions of IL-34 in the CNS are still elucidative. Here we show that CNS capillary endothelial cells also express CSF1R. IL-34 protected blood–brain barrier integrity by restored expression levels of tight junction proteins, which were downregulated by pro-inflammatory cytokines. The novel function of IL-34 on the blood–brain barrier may give us a clue for new therapeutic strategies in neuroinflammatory and neurodegenerative diseases such as multiple sclerosis and Alzheimer's disease.

## Introduction

Interleukin-34 (IL-34) has been identified as an additional ligand for colony stimulating factor-1 receptor (CSF1R), and it is broadly expressed in various organs including heart, brain, lung, liver, kidney, spleen, and colon [1]. IL-34 and colony stimulating factor-1/macrophage-colony stimulating factor (CSF-1/M-CSF) bind to the different regions of CSF1R and share no overt sequence homology [2]. Recent studies showed that IL-34 produced by epithelial lineage cells (e.g. keratinocytes, splenic vascular endothelial cells, and neurons) is necessary for the development of tissue macrophage-like cells (e.g. Langerhans cells, osteoclasts, and microglia) [3]–[5]. We also have shown that IL-34 is exclusively produced by neurons in CNS and induces proliferation of microglia [6]. We also showed that IL-34 attenuated the neurotoxic effect of oligomeric amyloid beta (Aβ) *in vitro* and intracerebroventricular administration of IL-34 ameliorates the impairment of associative learning in an AD mouse model [6]. Another study also demonstrated that IL-34 rescued neuronal damage in mouse models of AD and kinate-induced neurotoxicity [7]. These findings suggest distinct functions of IL-34 in the development of various CNS disorders. However, the precise functions of IL-34 in the CNS still remain to be elucidated.

The blood-brain barrier (BBB) is a tight seal composed of capillary endothelial cells, pericytes, and astrocytes [8]. The BBB contributes to maintenance of CNS homeostasis by limiting the entry of plasma components, erythrocytes, and immune cells from the circulating blood [9]–[11]. Tight junction (TJ) plays an important role in the barrier function of the BBB, which is composed by TJ proteins including claudins, occludin, and zonula occludens-1 (ZO-1) [12]. BBB disruption is frequently associated with synaptic and neuronal dysfunction in various neurological disorders such as multiple sclerosis (MS), AD, Parkinson's disease, and amyotrophic lateral sclerosis [13], [14]. Pro-inflammatory cytokines such as IL-1β, tumor necrosis factor-α (TNF-α), interferon-γ, and IL-17, are thought to downregulate the expression of tight junction proteins and contribute to the transmigration of inflammatory immune cells into the CNS, which exacerbates neuroinflammation in these diseases [14]–[19].

In this study, we found that the CNS capillary endothelial cells as well as microglia express CSF1R. We also showed that IL-34 restored pro-inflammatory cytokine–induced BBB disruption by upregulating the expression levels of tight junction proteins such as claudin-5 and occludin. These findings suggest the presence of neuronal regulation of BBB functions via IL-34, and upregulation of IL-34 in the CNS may be a novel therapeutic strategy against neuroinflammatory and neurodegenerative disorders.

## Materials and Methods

### Reagents

Recombinant mouse IL-1β, TNF-α, and IL-34 were purchased from R&D Systems (Minneapolis, MN, USA). The c-fms/CSF1R tyrosine kinase inhibitor GW2580 was used as a blocker of CSF1R signaling (Millipore, Bedford, MA, USA). Dylight 594–labeled tomato lectin was used as a capillary endothelial cell marker (Vector Laboratories, Burlingame, CA, USA).

### Animals

All protocols were approved by the Animal Experiment Committee of Nagoya University (approved number: 14018). C57BL/6J mice were purchased from Japan SLC (Hamamatsu, Japan).

### Cells

Primary neuronal cultures were prepared from the cortices of C57BL/6 mouse embryos at embryonic Day 17 as described previously [20]. Briefly, cortical fragments were dissociated into single cells in dissociation solution (Sumitomo Bakelite, Akita, Japan), and resuspended in neuron culture medium (Sumitomo Bakelite). Neurons were seeded onto 12-mm polyethylenimine-coated glass coverslips (Asahi Techno Glass Corp., Chiba, Japan) at a density of 5.0×10^4^ cells/well in 24-well culture plates and were incubated at 37°C in a humidified atmosphere containing 5% CO_2_. The purity of the cultures was >95% as determined by NeuN-specific immunostaining. Mouse brain capillary endothelial cell line MBEC4 [21] was maintained in Dulbecco's modified Eagle's medium supplemented with 10% fetal bovine serum. Confluent monolayer of MBEC4 cells was used as an established BBB model as described previously [22].

### BBB Permeability assay

The permeability of MBEC4 cell monolayers was measuring transendothelial electrical resistance (TER) as described previously [22]. Confluent monolayer of MBEC4 cells on the 24-well transwell inserts (3-µm pore size) were incubated with or without 20 ng/ml TNF-α, 20 ng/ml IL-1β, 0–100 ng/ml IL-34, or 1 µmol/L GW2580 for 24 h. TER was measured using a Millicell-ERS (Millipore). Resistances of blank filters were subtracted from those of filters with cells before final resistances (Ω • cm^2^) were calculated. Assays were carried out in five independent trials.

### Immunocytochemistry

Primary neurons and MBEC4 cells were fixed with 4% paraformaldehyde for 10 min, permeabilized using 0.1% Triton X-100 for 5 min, and blocked using 5% normal goat serum in phosphate-buffered saline (PBS) for 1 h at room temperature. Neurons were incubated with rabbit anti-mouse IL-34 polyclonal antibodies (ProSci, Poway, CA, USA), mouse anti-mouse microtubule-associated protein–2 (MAP-2) monoclonal antibody (Chemicon, Temecula, CA, USA) overnight at 4°C followed by a 1-h incubation with Alexa-conjugated secondary antibodies (Invitrogen, Carlsbad, CA, USA). MBEC4 cells were stained using rabbit anti-mouse CSF1R polyclonal antibodies (Abcam, Cambridge, UK) overnight at 4°C followed by a 1-h incubation with Alexa-conjugated secondary antibodies (Invitrogen). Nuclei were counterstained with Hoechst 33342 (Invitrogen). Images were analyzed using a deconvolution fluorescent microscope system (BZ-8000, Keyence, Osaka, Japan).

### Immunohistochemistry

Brains and lumbar spinal cords from C57BL/6J mice were fixed with 4% paraformaldehyde overnight, equilibrated in 20% sucrose with PBS for 48 hours, embedded in Tissue Tek O.C.T. compound (Sakura Finetechnical Co., Ltd., Tokyo, Japan), and frozen at −80°C overnight. Coronal brain sections and transverse spinal cord sections (20 µm-thick) were prepared using a cryostat. Sections were permeabilized using 0.3% Triton X-100 after blocking with 5% normal goat serum in PBS for 1 h. Sections were incubated with rabbit anti-mouse IL-34 polyclonal antibodies (ProSci), mouse anti-mouse MAP-2 monoclonal antibody (Chemicon), rabbit anti-mouse CSF1R polyclonal antibodies (Abcam), and Dylight 594–labeled tomato lectin (Vector Laboratories) overnight at 4°C followed by a 1-h incubation with Alexa-conjugated secondary antibodies (Invitrogen). Images were analyzed using a deconvolution fluorescent microscope system (BZ-8000, Keyence).

### RNA isolation and reverse transcription-polymerase chain reaction (RT-PCR)

MBEC4 cells were cultured at a concentration of 4×10^5^ cells/well in 24-well culture plates and stimulated with 100 ng/ml IL-34 for 24 h. Total RNA was extracted using the RNeasy Mini Kit (Qiagen, Valencia, CA). cDNAs encoding mouse IL-34 and glyceraldehyde 3-phosphate dehydrogenase (GAPDH) were generated by RT-PCR using SuperScript II (Invitrogen), Blend Taq DNA polymerase (Toyobo, Osaka, Japan), and the following specific primer sets:

CSF1R forward primer: 5′-AAGCAGAAGCCGAAGTACCA-3′

CSF1R reverse primer: 5′-GTCCCTGCGCACATATTTCAT-3′

GAPDH forward primer: 5′-TGTGTCCGTCGTGGATCTGA-3′

GAPDH reverse primer: 5′-CCTGCTTCACCACCTTCTTGA-3′

### Western Blotting

MBEC4 Cells were lysed in TNES buffer (50 mM Tris-HCl at pH 7.5, 150 mM NaCl, 1% Nonidet P-40, 2 mM EDTA, and 0.1% SDS) with protease inhibitor mixture (Complete Mini EDTA-free; Roche Diagnostics, Basel, Switzerland). Cell lysate proteins dissolved in Laemmli sample buffer (20 µg/well) were separated on 4–20% SDS-polyacrylamide gels (Mini-Protean TGX; Bio-Rad, Hercules, CA, USA) and transferred to Hybond-P polyvinylidene difluoride membranes (GE Healthcare, Piscataway, NJ, USA) as described previously [23]. The membranes were blocked for 1 h at room temperature with 5% skim milk in Tris-buffered saline containing 0.05% Tween-20, and then incubated overnight at 4°C with rabbit anti-mouse Zonula Occludens–1 (ZO-1) polyclonal antibodies, rabbit anti-mouse occludin polyclonal antibodies, rabbit anti-mouse claudin-5 polyclonal antibodies (Invitrogen), and mouse anti-β-actin monoclonal antibody (Sigma). After an overnight incubation with primary antibodies at 4°C, each blot was probed with horseradish peroxidase-conjugated anti-mouse IgG (GE Healthcare). Blots were then visualized with SuperSignal West Dura Extended Duration Substrate (Thermo Fisher Scientific, Waltham, MA, USA), and quantitated using a CS Analyzer 3.0 system (Atto, Tokyo, Japan). Assays were carried out in five independent trials.

### Statistical analysis

Statistical significance was analyzed with one-way analysis of variance followed by post-hoc Tukey's test, using GraphPad Prism version 5.0 (GraphPad Software, La Jolla, CA, USA).

## Results

### IL-34 is exclusively expressed in CNS neurons

**Figure 1:**
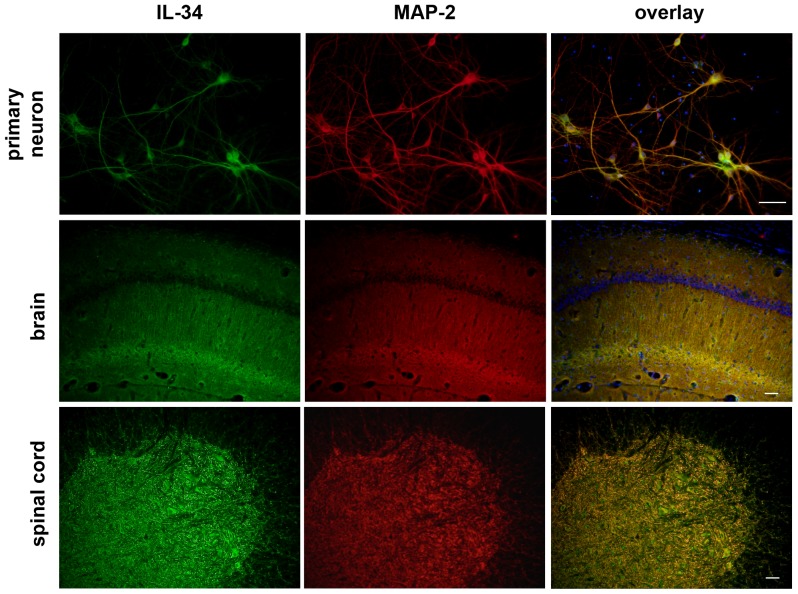
IL-34 is produced by neurons in the CNS. Immunofluorescence images of primary cortical neurons, brain sections, and lumbar spinal cord sections. Green, IL-34; red, MAP-2; blue, Hoechst nuclear counterstain. Scale bar, 50 µm.

In the previous study, we have reported that IL-34 protein is primarily expressed in neurons whereas IL-34 mRNA expression was detected in neurons and astrocytes [6]. First, we confirmed the expression pattern of IL-34 in the CNS using immunostaining in mouse primary cortical neurons, brains, and spinal cords. As shown in Fig. 1, IL-34 protein was exclusively expressed in neurons in the CNS.

### CNS capillary endothelial cells expressed IL-34 receptor CSF1R

**Figure 2:**
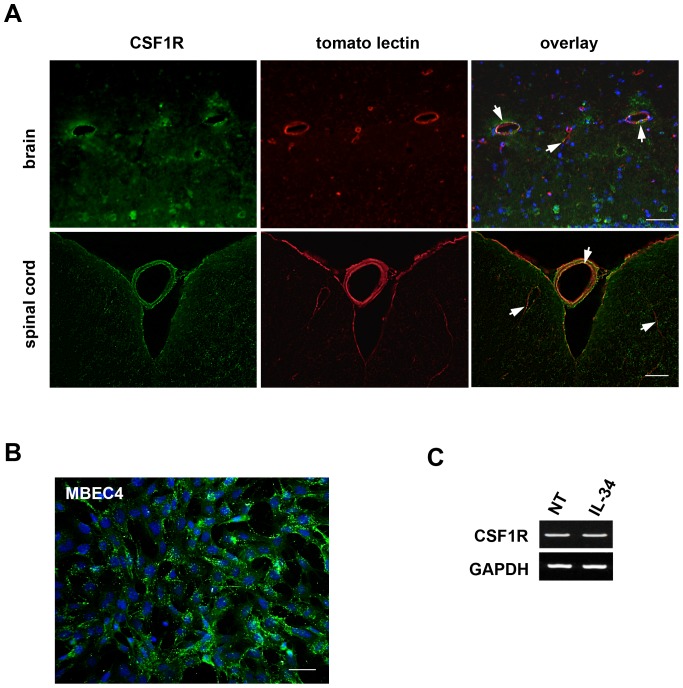
CNS endothelial cells express CSF1R. (A) Immunofluorescence images of brain sections, and lumbar spinal cord sections. Green, CSF1R; red, tomato lectin; blue, Hoechst nuclear counterstain. Arrows indicate CSF1R-immunopositivity in the capillary endothelial cells. Scale bar, 50 µm. (B) Immunofluorescence image of mouse brain capillary endothelial cell line MBEC4. Green, CSF1R; blue, Hoechst nuclear counterstain. Scale bar, 50 µm. (C) RT-PCR data for CSF1R. Stimulation with IL-34 did not alter the expression of CSF1R in MBEC4 cells.

Next, we examined the expression pattern of IL-34 receptor CSF1R protein in the CNS using immunostaining. In addition to microglia, CNS microvessels were also immunopositive for CSF1R (Fig. 2A, green). Its staining pattern in the microvessels was identical to that of tomato lectin (Fig. 2A, red and arrows in the overlap images) which selectively binds to the surface of capillary endothelial cells [24], suggesting that CNS capillary endothelial cells express CSF1R. Meninges and large vessel adventitia were also stained with CSF1R and tomato lectin. Although CSF1R has been detected on fibroblasts and smooth muscle cells which are the main components of meninges and adventitia [1], [7], the meninges and adventitia showed strong non-specific binding of antibodies and lectin. Therefore, the positive staining in meninges and adventitia may be artifact.

Furthermore, mouse brain capillary endothelial cell line MBEC4 cells strongly express CSF1R protein (Fig. 2B, green). MBEC4 cells constitutively express CSF1R mRNA, and stimulation with IL-34 did not alter CSF1R expression level (Fig. 2C). These data indicate that CNS capillary endothelial cells constitutively express CSF1R and are potential target of IL-34 in the CNS, as well as microglia.

### IL-34 restored BBB disruption via CSF1R signaling in endothelial cells

**Figure 3:**
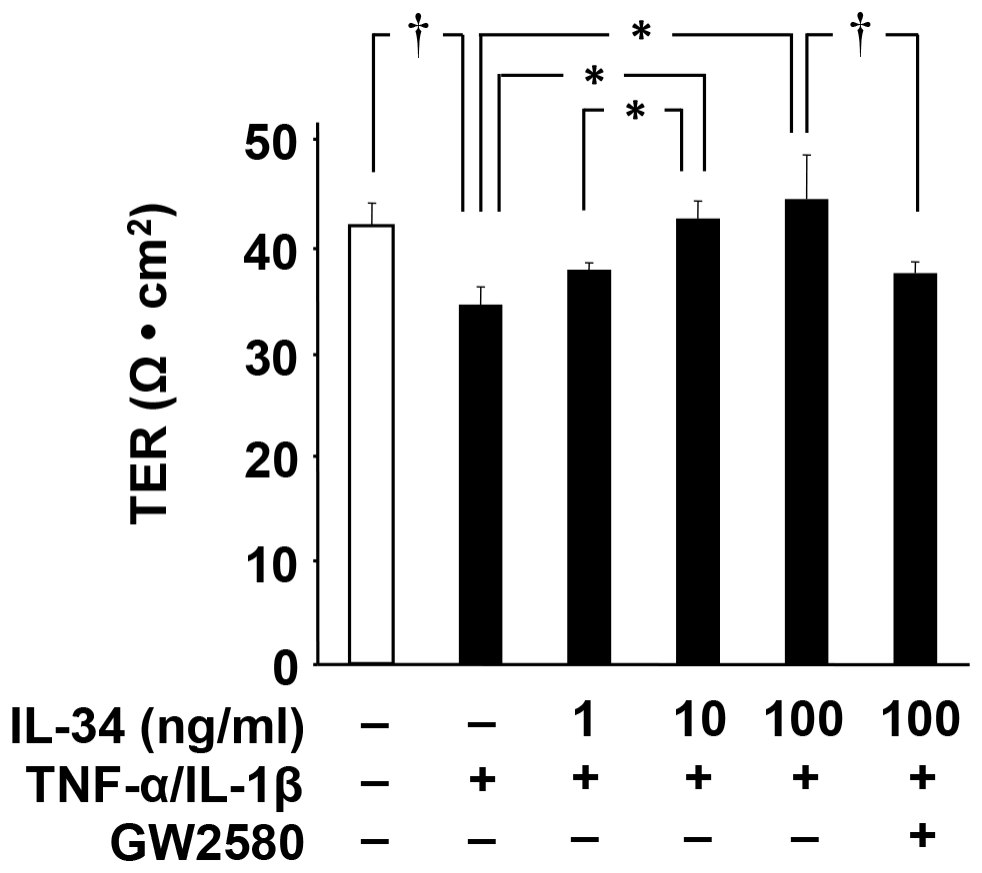
IL-34 restores damaged BBB integrity. MBEC4 cells were treated with TNF-α (20 ng/ml) and IL-1β (20 ng/ml) in the presence of IL-34 (0–100 ng/ml) and GW2580 (1 µmol/l). TER of MBEC4 cell monolayer was measured after a 24-h incubation. Values are means ± SEM (n = 5). *, *p*<0.05; †, *p*<0.01.

BBB disruption is a common pathological feature of various neurological diseases, and inflammatory cytokines such as IL-1β and TNF-α have been considered as causative factors that damage BBB integrity by downregulating TJ proteins in BBB endothelial cells [14]–[16], [25], [26]. To investigate whether IL-34 affects the BBB integrity, we evaluated BBB permeability by measuring TER in MBEC4 cell monolayer as an *in vitro* BBB model [22]. IL-34 significantly ameliorated a decrease in TER induced by IL-1β and TNF-α in a dose dependent manner (Fig. 3), whereas treatment with IL-34 alone did not alter untreated BBB integrity (data not shown). Moreover, addition of CSF1R signal inhibitor GW2580 ablated the effect of IL-34 on BBB (Fig. 3). These results indicate that IL-34 restored pro-inflammatory cytokine–mediated BBB disintegrity via CSF1R signaling in endothelial cells.

### IL-34 upregulated TJ proteins in BBB endothelial cells

**Figure 4:**
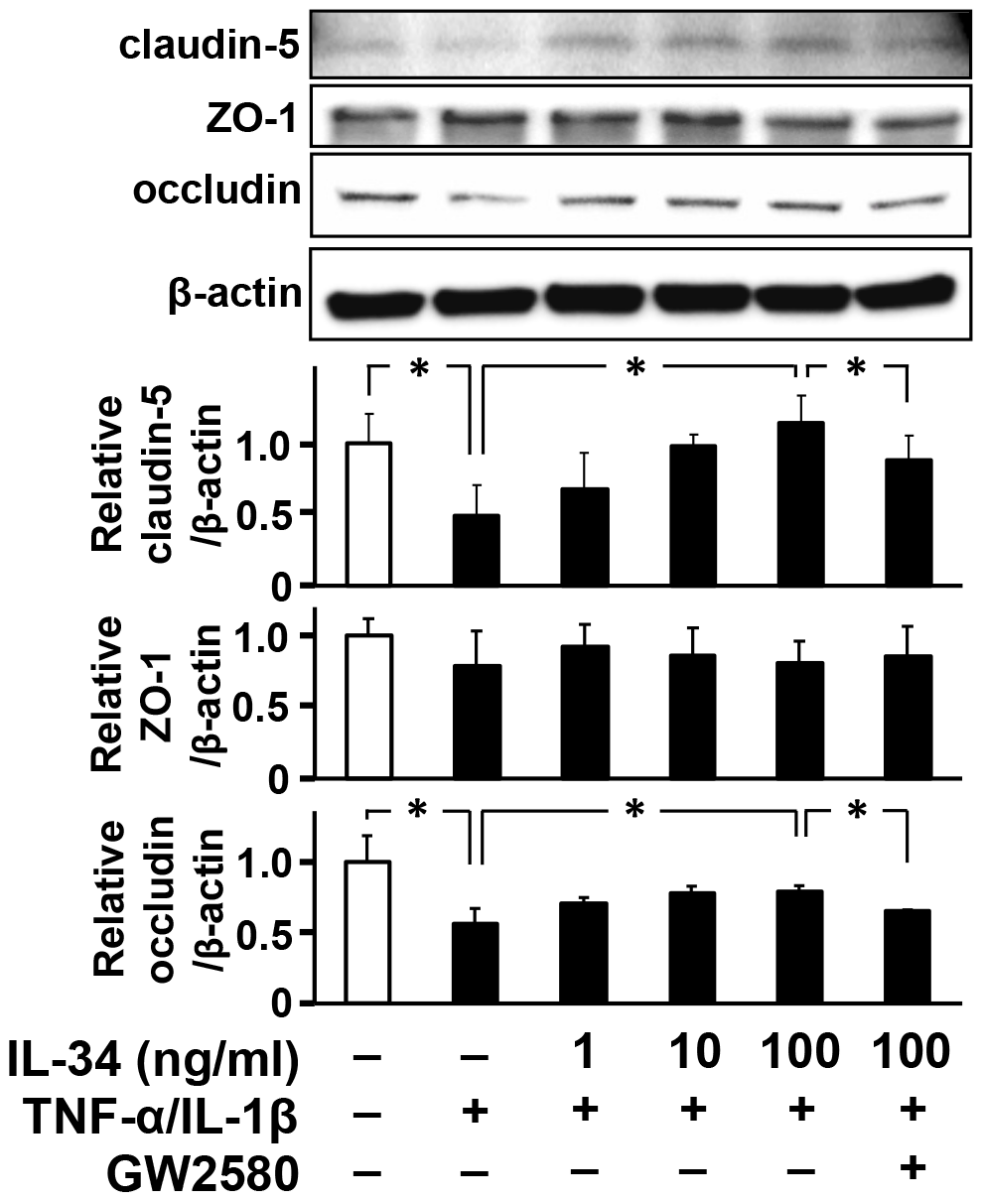
IL-34 upregulates tight junction proteins in MBEC4 cells. MBEC4 cells were incubated with TNF-α (20 ng/ml) and IL-1β (20 ng/ml) in the presence of IL-34 (0–100 ng/ml) and GW2580 (1 µmol/L) for 24 h. Upper, representative images of Western blots for tight junction proteins. Bottom, quantified expression levels of tight junction proteins relative to those in untreated cells. Values are means ± SEM (n = 5). *, *p*<0.05.

Next, we assessed whether IL-34 alters the expression levels of TJ proteins that are sensitive to pro-inflammatory cytokines [14]–[16]. Western blotting analysis detected that major TJ proteins such as claudin-5 and occludin were significantly downregulated by treatment with IL-1β and TNF-α (Fig. 4). Addition of IL-34 reversed the expression levels of these TJ proteins (Fig. 4), whereas treatment with IL-34 alone did not alter the expression levels of TJ proteins in untreated MBEC4 cells (data not shown). Addition of GW2580 canceled the effect of IL-34 on the expression of claudin-5 and occludin (Fig. 4). These data suggest that IL-34 rescues pro-inflammatory cytokine–induced BBB disruption via upregulating TJ proteins such as claudin-5 and occludin in BBB endothelial cells.

## Discussion

**Figure 5:**
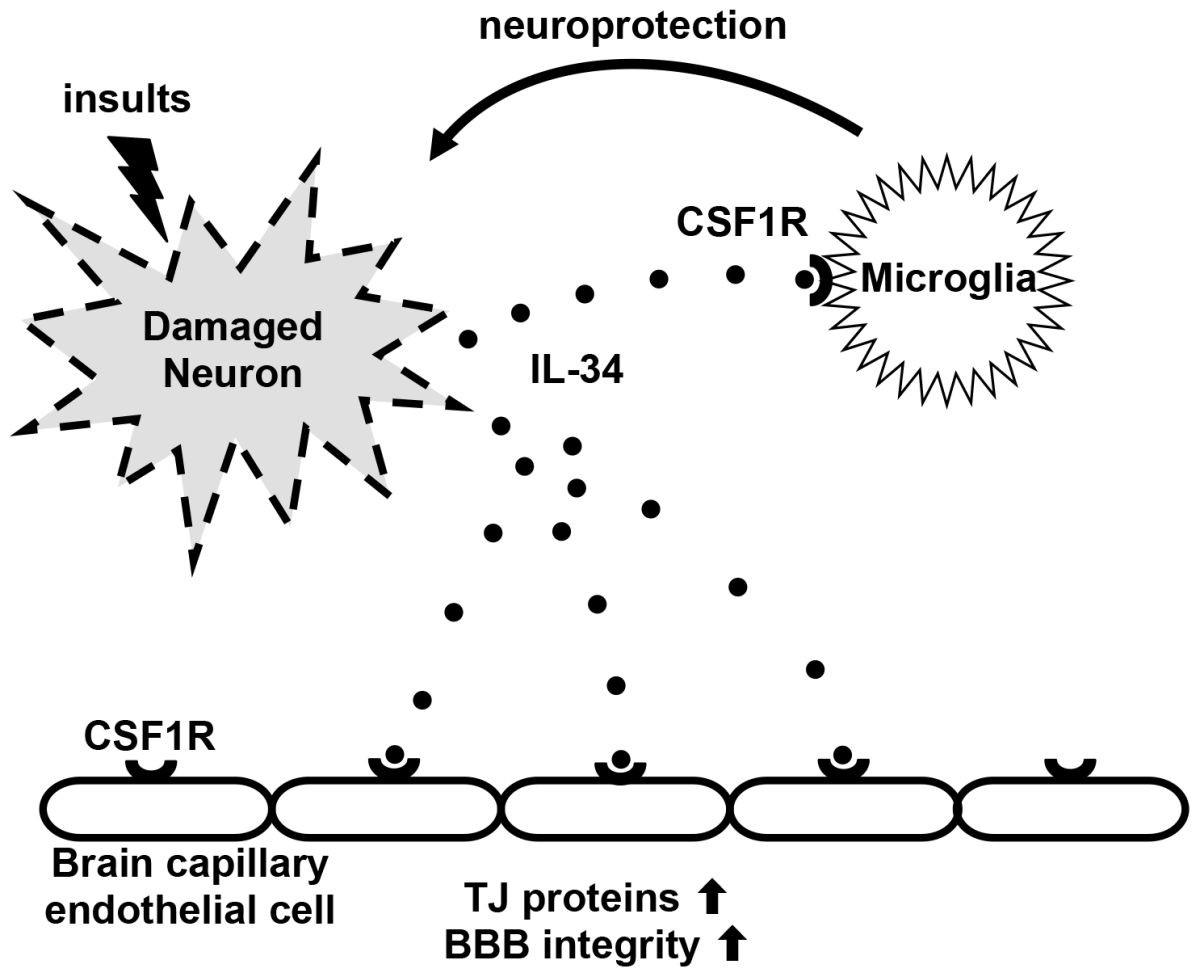
Model of the roles of IL-34 in the CNS. Damaged neurons secrete IL-34 as a “Help-me” signal. IL-34 binds its receptor CSF1R which is mainly expressed in microglia and BBB endothelial cells. CSF1R signaling enhances neuroprotection in microglia and restores BBB disruption by upregulating TJ proteins in capillary endothelial cells.

IL-34 is widely expressed in a variety of tissues including brain. Because IL-34 shares the same receptor with CSF-1/M-CSF, it has similar functions on monocyte lineage cells such as induction of proliferation and differentiation of macrophages, Langerhans cells, osteoclasts, and microglia [3]–[5], [27], [28]. In the CNS, IL-34 is mainly released by neurons, especially when they are damaged [6]. Like CSF-1/M-CSF, IL-34 induces microglial proliferation. In addition, IL-34 enhances microglial neuroprotective functions by inducing anti-oxidant enzyme heme oxigenase-1 (HO-1), and amyloid degrading enzyme insulin degrading enzyme (IDE). Moreover, we also found that IL-34 induces microglial production of TGF-β which negatively regulates microglial activation [29]. TGF-β dose-dependently suppressed microglial proliferation by IL-34 but attenuated oligomeric amyloid β–mediated neurotoxicity [29]. The neuroprotective functions of IL-34 was partially suppressed by blockade of TGF-β receptor signaling, suggesting that neuroprotective effect of IL-34 was in part mediated by microglial TGF-β production in response to IL-34. Thus, IL-34 released from damaged neurons acts as a “Help-me” signal which induces microglial neuroprotective effects with subsiding microglial activation (Fig. 5). The expression of CSF1R is reportedly high during early postnatal development, and is very low in adult brain [30]. IL-34 exhibited a broader regional expression than CSF-1/M-CSF, mostly without overlap, suggesting important role of IL-34–CSF1R signaling in regional neurogenesis. A previous study reported that CSF1R expression is increased in microglia of AD brains and microglia overexpressing CSF1R are neuroprotective [31]. Therefore, IL-34 produced by neurons [6] as well as CSF-1/M-CSF produced by astrocytes [32] may be involved in the development of neurodegenerative disesases such as AD via microglial CSF1R signaling.

In this study, we have shown that BBB endothelial cell expresses CSF1R and is a novel target of IL-34. BBB disruption has been implicated as a pathogenesis of various neurological disorders including MS and AD. A recent study showed that amyloid β suppressed expression of TJ protein ZO-1 in BBB endothelial cells via receptor for advanced glycation end products (RAGE) and claimed that amyloid β-RAGE interaction may be a potential molecular pathway in breakage of BBB integrity [33]. In addition, pro-inflammatory cytokines such as IL-1β, TNF-α, IFN-γ, and IL-17 have been considered as the candidates to increase BBB leakage [14]–[19]. Our findings revealed a novel function of IL-34–CSF1R signaling on the maintenance of BBB integrity via upregulating major TJ proteins claudin-5 and occludin in capillary endothelial cells (Fig. 5). A major downstream target of CSF1R signaling is cAMP responsive element-binding protein (CREB), which modulates the transcription of TJ proteins [34], [35]. Taken together, IL-34 released from damaged neurons may functions as a “Help-me” signal toward restoration of CNS homeostasis via microglia and BBB endothelial cells (Fig. 5). Our study clarified the presence of neuronal regulation of BBB functions via IL-34–CSF1R signaling. IL-34–CSF1R pathway may be novel therapeutic target for neuroinflammatory and neurodegenerative disorders such as MS and AD.
